# Automated Computational Detection of Disease Activity in ANCA-Associated Glomerulonephritis Using Raman Spectroscopy: A Pilot Study

**DOI:** 10.3390/molecules27072312

**Published:** 2022-04-02

**Authors:** Adam D. Morris, Daniel L. D. Freitas, Kássio M. G. Lima, Lauren Floyd, Mark E. Brady, Ajay P. Dhaygude, Anthony W. Rowbottom, Francis L. Martin

**Affiliations:** 1 Renal Medicine, Royal Preston Hospital, Preston PR2 9HT, UK; lauren.floyd@lthtr.nhs.uk (L.F.); mark.brady@lthtr.nhs.uk (M.E.B.); ajay.dhaygude@lthtr.nhs.uk (A.P.D.); 2 Institute of Chemistry, Biological Chemistry and Chemometrics, Federal University of Rio Grande do Norte, Natal 59072-970, Brazil; daniellucas77@hotmail.com (D.L.D.F.); kassiolima@gmail.com (K.M.G.L.); 3 Department of Immunology, Royal Preston Hospital, Preston PR2 9HT, UK; anthony.rowbottom@lthtr.nhs.uk; 4 School of Medicine, University of Central Lancashire, Preston PR1 2HE, UK; 5 Biocel Ltd., Hull HU10 7TS, UK

**Keywords:** ANCA, ANCA-associated, vasculitis, glomerulonephritis, Raman spectroscopy

## Abstract

Biospectroscopy offers the ability to simultaneously identify key biochemical changes in tissue associated with a given pathological state to facilitate biomarker extraction and automated detection of key lesions. Herein, we evaluated the application of machine learning in conjunction with Raman spectroscopy as an innovative low-cost technique for the automated computational detection of disease activity in anti-neutrophil cytoplasmic autoantibody (ANCA)-associated glomerulonephritis (AAGN). Consecutive patients with active AAGN and those in disease remission were recruited from a single UK centre. In those with active disease, renal biopsy samples were collected together with a paired urine sample. Urine samples were collected immediately prior to biopsy. Amongst those in remission at the time of recruitment, archived renal tissue samples representative of biopsies taken during an active disease period were obtained. In total, twenty-eight tissue samples were included in the analysis. Following supervised classification according to recorded histological data, spectral data from unstained tissue samples were able to discriminate disease activity with a high degree of accuracy on blind predictive modelling: F-score 95% for >25% interstitial fibrosis and tubular atrophy (sensitivity 100%, specificity 90%, area under ROC 0.98), 100% for necrotising glomerular lesions (sensitivity 100%, specificity 100%, area under ROC 1) and 100% for interstitial infiltrate (sensitivity 100%, specificity 100%, area under ROC 0.97). Corresponding spectrochemical changes in paired urine samples were limited. Future larger study is required, inclusive of assigned variables according to novel non-invasive biomarkers as well as the application of forward feature extraction algorithms to predict clinical outcomes based on spectral features.

## 1. Introduction

Anti-neutrophil cytoplasmic autoantibody (ANCA)-associated vasculitis (AAV) is a complex auto-immune disease that typically causes multi-organ and life-threatening disease. It results from the necrotising inflammation of small- and medium-sized blood vessels which characteristically lack any significant immune complex deposits on histopathology. Renal involvement with ANCA-associated glomerulonephritis (AAGN) tends to present with rapidly progressive disease and often denotes a higher mortality risk compared to patients without renal disease, particularly amongst those with dialysis dependence [1,2,3]. Since its initial description in the early 1980s, patient outcomes have improved in parallel with advancing treatment strategies, with a substantial proportion of patients receiving remission-induction therapy and achieving dialysis independence by four months [4,5]. However, AAV is often characterised by a relapsing disease course and determining if changes in renal function or urinalysis are attributable to active disease without a repeat biopsy or a reliable peripheral biomarker presents a diagnostic challenge, particularly in renal limited disease with no other clinical cues.

Renal biopsy remains the gold standard for the diagnosis of AAGN, but its serial use for disease monitoring is restricted by the inherent procedural risks and resource requirements. The Berden classification system categorises histopathological findings in AAGN into four key subgroups: focal disease (≥50% normal glomeruli), crescentic disease (≥50% cellular crescents), sclerotic disease (≥50% globally sclerotic glomeruli) and mixed disease with no predominant lesion [6]. Since its initial description in 2010, it has been validated by numerous studies and has been shown to be of predictive clinical value, with poorer renal function amongst those with a higher degree of sclerosis and more favourable outcomes in those with a focal class of disease [6,7,8,9,10,11]. Although previously reported outcomes varied amongst crescentic and mixed class disease, a recent meta-analysis found no significant difference in end stage kidney disease between the two groups [11]. Emerging from these studies was the importance of the percentage of normal glomeruli as a significant predictor of renal survival, as well as the degree of tubulointerstitial disease [7,8,9,10]. T-cell mediated tubilitis and, as expected, a higher degree of interstitial fibrosis and tubular atrophy (IFTA) are associated with poorer renal outcomes, with the degree of IFTA increasing in parallel with the degree of glomerular scarring [8,9,10]. These features have since been incorporated into two predictive models: the Mayo chronicity score and the ANCA renal risk score [3,12]. The former accounts for glomerulosclerosis, IFTA, and aterioslcerosis with a lower likelihood of renal recovery associated with a higher score [3]. The ANCA renal risk score accurately predicts the risk of end stage renal disease by using a graded score for renal function at presentation, the degree of IFTA and the proportion of normal glomeruli [11,12].

The ability to non-invasively determine the prevailing histopathological lesion in real time would prove to be an invaluable tool to aid disease monitoring in renal limited AAV. Furthermore, it could facilitate individualised therapy, helping to identify those least likely to benefit from continued or intensified therapy in order to mitigate any potential treatment related harm. This study aims to differentiate spectral data obtained from renal tissue according to key histological lesions in cases of AAGN to evaluate the role of Raman spectroscopy as a method for automated computational detection of disease activity. Additionally, subsequent comparative analysis of the spectral data from paired urine samples at the time of renal biopsy will explore the role of biospectroscopy as a non-invasive surrogate marker of histological activity in renal vasculitis, in effect providing a liquid biopsy.

## 2. Materials and Methods

### 2.1. Patients & Ethics

From February to August 2019, consecutive patients with active AAGN and those in disease remission were recruited. For those in disease remission at the time of recruitment, where available, archived renal tissue samples taken at the time of initial diagnosis and held by the tissue bank at Royal Preston Hospital were obtained. For those patients with active disease at the time of recruitment, renal tissue samples along with paired urine samples taken immediately prior to renal biopsy were collected. The definition of AAV as outlined by the 2012 Chapel Hill Consensus Conference was used. Patients who did not meet this criterion, who were aged < 18 years, unable to provide consent, or exhibited dual positivity with anti-glomerular basement membrane disease were excluded. All participants were registered with the Department of Renal Medicine regional vasculitis service at Lancashire Hospitals NHS Foundation Trust, Preston, UK. Informed written consent was obtained prior to enrollment in accordance with study approval from the Health Research Authority, Cambridge South Research Ethics Committee (REC reference 18/EE/0194) and the Research and Development team in the Centre for Health Research and Innovation at Lancashire Teaching Hospitals NHS Foundation Trust.

Baseline clinical assessment comprised of participant demographics, histological evaluation of renal biopsies by a renal pathologist, and salient laboratory results at the time of biopsy including ANCA serotype, serum creatinine and urine protein creatinine ratio. Recorded histological data included the assigned Berden classification (focal: ≥50% normal glomeruli, crescentic: ≥50% cellular crescents, sclerotic: ≥50% globally sclerotic glomeruli, and mixed: no predominant lesion) [6], the percentage of normal glomeruli (N0 > 25%, N1 10–25%, N2 < 10%), the severity of interstitial fibrosis and tubular atrophy (IFTA) (T1 > 25%, T0 ≤ 25%), and the presence of interstitial infiltrate, necrotising glomerular lesions, extra-glomeruli arteritis, and vessel wall necrosis. The percentage of normal glomeruli and degree of IFTA was assessed according to the grading scale applied by Brix et al., in the ANCA renal risk score [12]. Urine samples were sent for microscopy and culture to determine the presence of bacteriuria and its potential impact as a confounding factor on spectroscopic analysis.

### 2.2. Sample Collection & Preparation

Following their initial acquisition and departmental assessment, formalin-fixed paraffin-embedded tissue blocks were retrieved from the tissue bank at Royal Preston Hospital. To ensure close correlation between histopathology reports and spectroscopic measurements, contiguous sections of 10 μm thickness were used from each tissue block. After sections had been cut and placed on IR-reflective aluminium coated FisherBrand^TM^ (Loughborough, UK) slides, all samples were deparaffinised according to local protocols using xylene and ethanol in order to avoid any potential impact on the spectral data obtained.

Urine samples were collected in reagent-free, sterile containers and centrifuged at 3000 rpm, 4 °C for 10 min. The resulting supernatant urine was collected in 0.5 mL Eppendorf tubes and stored at −80 °C. When required for experimentation, samples were thawed at room temperature, after which 30 μL aliquots were placed on IR-reflective aluminium-coated FisherBrand^TM^ slides and left to air dry for a minimum of 2 h prior to spectroscopic analysis.

### 2.3. Spectral Acquisition

Spectral data was obtained using a Renishaw InVia Raman spectrometer in conjunction with a charge-coupled device and Leica confocal microscope (Renishaw pls UK). This system utilised a 200 mW laser diode at a wavelength of 785 nm with a grating of 1200 lines/mm. Renishaw WiRE^TM^ was used to control data acquisition. The spectral range was set between 400–2000 cm^−1^ with a 1 cm^−1^ spectral resolution. For tissue mapping, spectral data was obtained over the entirety of a 1000 × 500 μm^2^ acquisition area, using 5× magnification, 100% laser power, exposure time of 0.1 s with 5 × 5 steps in high confocality and spectral centre of 1300 cm^−1^. Three select regions of interest where the highest number of glomeruli were visible within the acquisition area were analysed from each sample. For urine samples, ten individual spectral points were taken per sample over an acquisition area of 250 × 125 μm^2^ using 20× magnification, 10% laser power, and an exposure time of 10 s, with an extended grading scale. Within each acquisition area, four spectral points were taken along the superior horizontal plane, four along the inferior horizontal plane, and two in the middle. For both tissue and urine samples, one representative mean spectrum was subsequently generated per sample for later use in the analysis.

### 2.4. Spectral Pre-Processing

Three-dimensional (3D) Raman mapping images were uploaded into MATLAB R2014b environment (MathWorks Inc., Natick, MA, USA) and unfolded into two-dimensional (2D) structures containing *n* rows (number of spectra) and *m* columns (number of wavenumbers). Initially raw spectral data obtained from both tissue and urine samples were evaluated for anomalous spectra or biased patterns. Pre-processing was then undertaken, applying mathematical techniques to remove or reduce chemical signals that are not relevant to the analyte target property or sample discrimination in order to improve the precision of any qualitative and quantitative analysis [13]. The raw data obtained for both tissue and urine samples were then submitted to the same procedures; spectral data was cut in the region of 800–1800 cm^−1^ with application of Savitzky–Golay (SG) 2nd order derivative (51 window points, 2nd order polynomial) and vector normalization to correct for random noise, baseline distortions and physical difference between samples [14]. All resulting pre-processed data was mean-centred prior to model construction for discriminant analysis. All models were trained and tested with pre-processed data only.

### 2.5. Multivariate Analysis

In order to identify any natural clustering patterns or trends in the pre-processed data, principal component analysis (PCA) is a multivariate technique that was used for initial exploratory analysis and data reduction. In this process, the initial spectral wavenumber variables are reduced to a few principal components (PC) responsible for the majority of the original data variance. Each PC is composed of scores and loadings. The scores represent the variance on sample direction, therefore being used to identify similarity/dissimilarity patterns between the samples, whereas the loadings represent the variance on the variable direction, therefore being used to identify possible spectral markers responsible for the patterns observed and any potential class separation on the scores plot.

In each model, the spectral data from both tissue and urine samples was segregated according to the presence of recorded histological data to generate the experimental classes of the assigned Berden classification (focal, crescentic, sclerotic, and mixed) [6], the percentage of normal glomeruli (N0 > 25%, N1 10–25%, N2 < 10%), the severity of IFTA (T1 > 25%, T0 ≤ 25%) and the presence of interstitial infiltrate, necrotising glomerular lesions, extra-glomeruli arteritis, and vessel wall necrosis. A lower proportion of normal glomeruli indicates greater disease burden with their composition guiding the degree of acute disease vs. chronicity. A higher burden of IFTA and sclerosed glomeruli represent chronic damage, whereas the remaining lesions described are indicative of active disease. An experimental class according to ANCA seropositivity and, in positive cases, ANCA serotype were also generated. For each, the total data obtained were used to build the models, without dividing samples by selection methods due to the limited sample availability in certain classes. The models were evaluated using the Venetian blinds cross-validation method. Analysis of the spectral mean was used as the test set for blind predictive modelling. These test samples are independent from training data as they are not used in the model training process and are considered new data to the model. The overall performance of each discriminant analysis algorithm was then compared.

### 2.6. Chemometric Models

The discriminant analysis algorithms of principal component analysis linear discriminant analysis (PCA-LDA), partial least squares discriminant analysis (PLS-DA), support vector machines (SVM), and genetic algorithm linear discriminant analysis (GA-LDA) were subsequently applied to the pre-processed data for supervised classification. Models were constructed using the PLS Toolbox and Classification Toolbox graphical user interface of the Milano Chemometrics group [15].

PLS-DA is a well-established chemometric technique for supervised classification. For this process, the dataset was broken down into a few latent variables (LVs) responsible for maximizing the covariance between the spectral data and the response information, which in this the case is the histological or serological category. The number of latent variables was determined by the leave-one-out type cross-validation to prevent overfitting. The key distinguishing spectral peaks were then identified using the PLS-DA coefficient.

While PCA and PLS perform a reduction in the number of original variables generating another set of variables, the genetic algorithm (GA) selects the most important variables based on a selection, recombination, and mutation of a set of the original variables. Thus, its main objective is to reduce the number of variables, taking advantage of not changing the type of variable and original information according to an adjustment function. The GA routine was carried out during 100 generations with 200 chromosomes each. Mutation and crossover probabilities were adjusted to 10% and 60% respectively. The best solution set for this algorithm is based on the fitness value. The adjustment function is calculated as the inverse of the cost function G, which can be defined as follows:

(1) $$\;G=\frac{1}{N_{V}}\overset{N_{V}}{\underset{n=1}{\sum }}g_{n}$$

where $N_{V}$ is the number of validation samples and $g_{n}$ is defined as follows:

(2) $${\;g}_{n}=\frac{r^{2}\left(x_{n},{\;m}_{I\left(n\right)}\right)}{\min_{I\left(m\right)\neq I\left(n\right)}r^{2}\left(X_{n},{\;m}_{I\left(m\right)}\right)}$$

where the numerator is the squared Mahalanobis distance between object $x_{n}$ of class index I(n) and the sample mean $m_{I\left(n\right)}$ of its true class; and the denominator is the squared Mahalanobis distance between object x(n) and the centre of the closest wrong class.

The classifiers used here were LDA and SVM. LDA is based on the Mahalanobis distance between samples and considers that all classes have a similar variance structure, building a model based on pooled covariance matrix. The input data used for LDA are scores obtained via PCA. The LDA classifier, non-Bayesian form, can be obtained by the following equation for a sample i in a given class k:

(3) $${\;L}_{\mathrm{ik}}={\left(x_{i}-{\bar{x}}_{k}\right)}^{T}C_{\mathrm{pooled}}^{-1}\left(x_{i}-{\bar{x}}_{k}\right)$$

where $x_{i}$ is a vector with variables for sample i; ${\bar{x}}_{k}$ is the mean of class k; and $C_{\mathrm{pooled}}$ is the pooled covariance matrix between the classes.

SVM is a machine learning technique that uses the kernel transformation [16]. This projects data in a non-linear fashion into a feature dimension to provide the radial basis function (RBF) and classify samples according to a linear threshold. This has the advantage of being able to adjust for different data distributions to provide a more powerful discriminant analysis but may carry a higher risk of overfitting. The RBF is calculated as follows:

(4) $$\;k\left(x_{i},z_{j}\right)=\exp\left(-\gamma \left|\left|x_{i}-z_{j}^{2}\right|\right|\right)\;$$

where $x_{i}$ and $z_{j}$ are sample measurements vectors and γ is a tuning parameter that controls the RBF width. In the RBF function, the γ parameter was set to 1. The SVM classification is obtained by the following equation:

(5) $$\;f\left(x\right)=\mathrm{sign}\left(\overset{N_{\mathrm{SV}}}{\underset{i=1}{\sum }}\alpha_{i}y_{i}k\left(x_{i},z_{j}\right)+b\right)$$

where $N_{\mathrm{SV}}$ is the number of support vectors; $\alpha_{i}$ is the Lagrange multiplier; $y_{i}$ is the class membership, ranging from −1 to +1; $k\left(x_{i},z_{j}\right)$ is the kernel function and b is the bias parameter. The parameters used were obtained through an internal validation dataset.

### 2.7. Model Validation

The calculation of accuracy, sensitivity, specificity, F-scores, and G-scores were calculated for the test set for model validation. Accuracy represents the total number of samples correctly classified, considering true and false negatives. The sensitivity represents the proportion of positive samples correctly classified and the specificity represents the proportion of negative samples correctly classified. The F-score measures the models performance considering imbalanced data, whereas the G-score is a metric that is used to evaluate the models performance independent of class size [17]. The statistical parameters presented can be calculated as follows:

(6) $$\mathrm{Accuracy}\;\left(\mathrm{AC}\right)=\left(\frac{\mathrm{TP}\;+\;\mathrm{TN}}{\mathrm{TP}\;+\;\mathrm{FP}\;+\;\mathrm{TN}\;+\;\mathrm{FN}}\right)\times 100$$

(7) $$\mathrm{Sensitivity}\;\left(\mathrm{SENS}\right)=\left(\frac{\mathrm{TP}}{\mathrm{TP}\;+\;\mathrm{FN}}\right)\times 100$$

(8) $$\mathrm{Specificity}\;\left(\mathrm{SPEC}\right)=\left(\frac{\mathrm{TN}}{\mathrm{TN}\;+\;\mathrm{FP}}\right)\times 100$$

(9) $$F\text{-}\mathrm{Score}\;\left(\mathrm{Fs}\right)=\left(\frac{2\times \;\mathrm{SENS}\;\times \;\mathrm{SPEC}}{\mathrm{SENS}\;+\;\mathrm{SPEC}}\right)\times 100$$

(10) $$G\text{-}\mathrm{Score}\;\left(\mathrm{Gs}\right)=\left(\sqrt{\mathrm{SENS}\;\times \;\mathrm{SPEC}}\right)\times 100$$

where FN stands for false negative, FP for false positive, TP for true positive, TN for true negative, AC for accuracy, SENS for sensitivity, SPEC for specificity, Fs for F-score, and Gs for G-Score. Herein, although both were derived from the same experiment, the test samples are independent from the training samples. The validation performance depicted here are ideal for small datasets in order to have a good approximation of the real blind performance [13]. For further model validation, it would be necessary to realise a second experiment with completely new samples in order to assess the blind model performance.

## 3. Results

### 3.1. Study Population

Over the 6-month study period 28 patients were recruited for the present study; 11 with new presentation AAGN and 17 currently in disease remission. One patient was excluded due to the development of dual positivity with anti-glomerular basement autoantibodies. Table 1 outlines the characteristics of the overall study population. Recently processed and archived renal tissue samples taken at the time of active disease were obtained for all remaining 27 participants, with paired urine samples prior to biopsy in all ten cases of newly presenting AAGN.

Amongst those participants with a paired urine sample at the time of renal biopsy (n = 10), mean age was 63 ± 7.6 with 80% (n = 8) female predominance, median serum creatinine of 282 μmol/L (IQR 447–201) and 90% (n = 9) seropositivity; four with anti-MPO and five with anti-PR3 associated disease. The mean number of glomeruli per biopsy sample was 19 ± 9, with a distribution of disease of 50% (n = 5) focal, 40% (n = 4) mixed and 10% (n = 1) crescentic according to the Berden classification system [6]. The proportion of samples with >25% normal glomeruli (grade N0) was 70% (n = 7) and 30% (n = 3) exhibited <10% (grade N2) normal glomeruli. A similar distribution for IFTA was seen; 70% (n = 7) ≤25% (grade T0) and 30% (n = 3) >25% (grade T1). The observed frequency of necrotising glomerular lesions and interstitial infiltrate were 30% (n = 3). For extra-glomerular arteritis and vessel wall necrosis 20% (n = 2) were affected. The median uPCR and urine white cell count was 89 mg/mmol (IQR 258-63) and 31 × 10^9^/L (IQR 34-27) respectively. None of the collected urine samples displayed any bacterial growth.

### 3.2. Spectral Data & Classification Models: All Renal Tissue Samples

**Figure 1:**
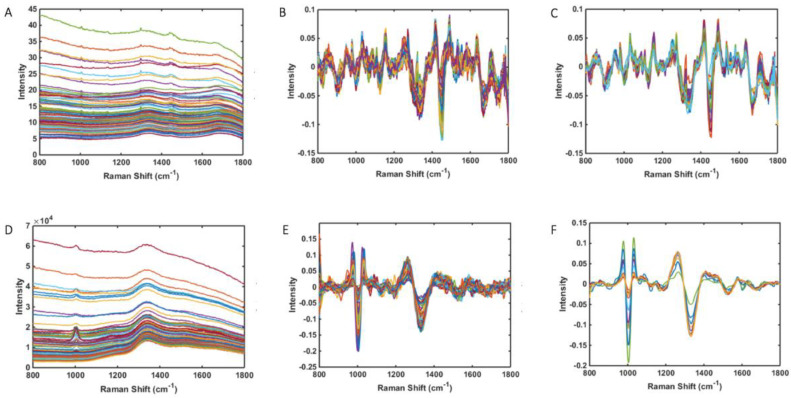
**Raman spectral data—**(**A**) Total raw spectra for all tissue samples (n = 81) (**B**) Total pre-processed spectra for all tissue samples (n = 81) (**C**) Average pre-processed spectra for all tissue samples (n = 27) (**D**) Total raw spectra for paired urine samples (n = 100) (**E**) Pre-processed spectra for paired urine samples (n = 100) (**F**) Average pre-processed spectra paired urine tissue samples (n = 10).

For the three spectra obtained from each sample image, one representative mean spectrum was generated per sample. As such, there are a total of 81 spectra for the 27-sample cohort and consequently 27 representative mean spectra. The total raw spectra, total pre-processed spectra and average pre-processed spectral data for the overall study population are shown in Figure 1A, B and C, respectively. For the construction of supervised classification models, both the total raw spectra and pre-processed spectra were used as training data with known categories according to each experimental class. Following cross-validation using the leave-one-out approach, the mean spectral data was applied as the test set for blind predictive modelling to validate the classification systems performance. In this construct, the cross-validation data is the most significant result that should be considered, representing the model’s ability to correctly predict new data based on the existing knowledge obtained from any training data. This process helps to mitigate any potential overfitting. Due to the unbalanced sample size distribution amongst all four Berden classes, comparative analysis was only feasible between focal (n = 15) and mixed (n = 9) disease. Similarly, evaluation of normal glomeruli was undertaken as a sample distribution of those with >25% normal glomeruli (group N0) (n = 21) vs. those exhibiting <25% normal glomeruli (groups N1 & N2) (n = 6).

**Figure 2:**
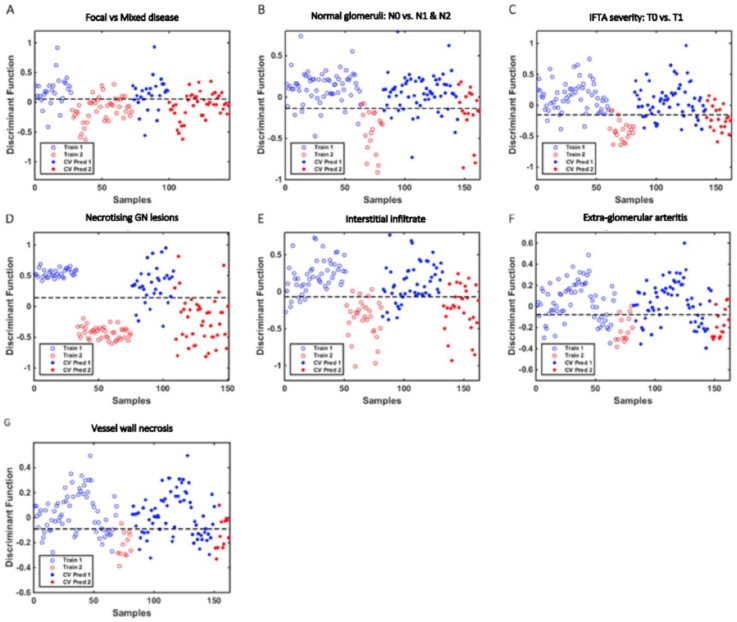
**PLS-DA discriminant function graphs for the classification of histological data using spectral data from all tissue samples with corresponding receiver operating characteristic curve data. Train 1 and Train 2 represent training sample data for each histological group. CV Pred 1 and CV Pred 2 represent test sample data for each histological group analysed by the cross-validation (CV) prediction process:** (**A**) Mixed vs. focal disease (train 1 & CV Pred 1 vs. train 2 & CV Pred 2) area under ROC 0.85 (**B**) Normal glomeruli N0 vs. N1 & N2 (train 1 & CV Pred 1 vs. train 2 & CV Pred 2) area under ROC 0.96 (**C**) Interstitial fibrosis and tubular atrophy (IFTA) severity T0 vs. T1 (train 1 & CV Pred 1 vs. train 2 & CV Pred 2) area under ROC 0.98 (**D**) Necrotising glomerular (GN) lesions absent vs. present (train 1 & CV Pred 1 vs. train 2 & CV Pred 2) area under ROC 1 (**E**) Interstitial infiltrate absent vs. present (train 1 & CV Pred 1 vs. train 2 & CV Pred 2) area under ROC 0.97 (**F**) Extraglomerular arteritis absent vs. present (train 1 & CV Pred 1 vs. train 2 & CV Pred 2) area under ROC 0.89 (**G**) Vessel wall necrosis absent vs. present (train 1 & CV Pred 1 vs. train 2 & CV Pred 2) area under ROC 0.92.

**Figure 3:**
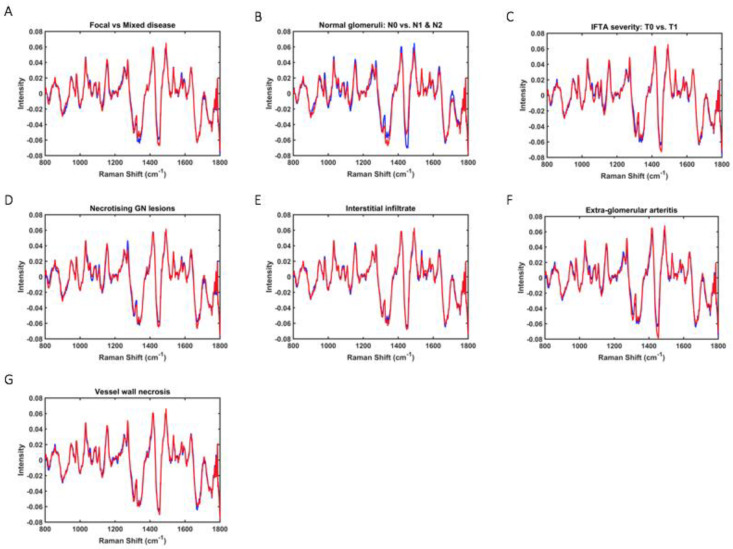
**Mean Raman spectral data for each histological group—**(**A**) focal vs. mixed disease, (**B**) proportional of normal glomeruli, (**C**) severity of interstitial fibrosis and tubular atrophy (IFTA), (**D**) presence of necrotising glomerular (GN) lesions, (**E**) presence of interstitial infiltrate, (**F**) presence of extra-glomerular arteritis, (**G**) presence of vessel wall necrosis.

PLS-DA discriminant function graphs and the classification model performance according to histological data for all renal biopsy samples are shown in Figure 2 and Table 2, respectively. The mean Raman spectral data for each histological group is shown in Figure 3. The spectral profiles for necrotising glomerular lesions, interstitial infiltrate, and IFTA yielded the most accurate results. This is evident with an F-score 95% for >25% interstitial fibrosis and tubular atrophy (sensitivity 100%, specificity 90%, area under ROC 0.98), 100% for necrotising glomerular lesions (sensitivity 100%, specificity 100%, area under ROC 1), and 100% for interstitial infiltrate (sensitivity 100%, specificity 100%, area under ROC 0.97). The predictive performance in distinguishing focal from mixed disease, >25% normal glomeruli, and the presence of vessel wall necrosis was limited with a sensitivity of <60% in each model. Similarly, the discriminant model for ANCA was not significant with a sensitivity of only 56% in seropositive cases.

### 3.3. Spectral Data and Classification Models: Comparative Results for Tissue & Paired Urine Samples

Based on the findings observed in the overall cohort, a comparative subgroup analysis was undertaken amongst those with a paired urine sample at the time of renal biopsy (n = 10). This sought to determine if equally good discrimination for necrotising glomerular lesions, interstitial infiltrate, and >25% IFTA could be demonstrated in both biosamples. For the three spectra obtained from each tissue sample, one representative mean spectrum was generated per sample, resulting in a total of 30 spectra and 10 representative mean spectra for the subgroup. Ten individual spectral points were obtained from each urine sample, generating a total of 100 spectra and 10 representative average spectra. Findings are shown in Table 3 and exhibit limited accuracy in distinguishing the presence of each category in urine on blind predictive modelling with a sensitivity < 60% and F-score < 65% for each. Subgroup model data for normal glomeruli, Berden classification, vessel wall necrosis, and extra-glomerular arteritis are not presented in view of their suboptimal performance in tissue analysis amongst the overall cohort.

### 3.4. Key Discriminating Spectral Biomarkers: Tissue & Paired Urine Samples

**Figure 4:**
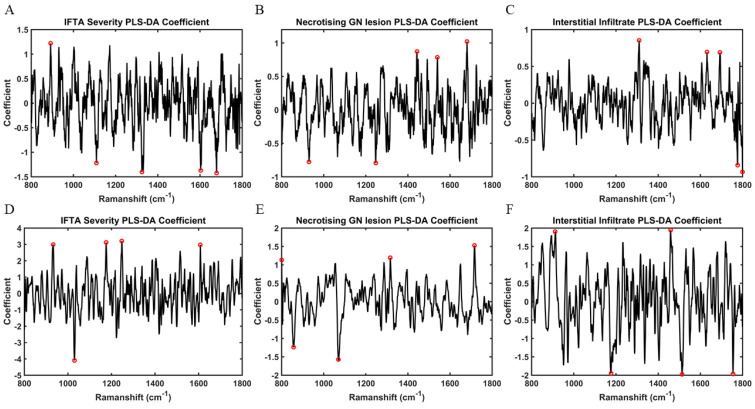
**PLS-DA coefficients for identification of spectral biomarkers in tissue and corresponding paired urine samples (n = 10)**—(**A**) Wavenumber variables associated with interstitial fibrosis & tubular atrophy (IFTA) in tissue samples—891.92 cm^−1^ (saccharide band) (**B**) Wavenumber variables associated with necrotising glomerular (GN) lesions in tissue samples—1680 cm^−1^ (C=O, stretching vibrations of cortisone), 1443 cm^−1^ (CH2 bending mode of proteins & lipids CH2 deformation), 1539 cm^−1^ (amide carbonyl group vibrations & aromatic hydrogens) (**C**) Wavenumber variables associated with interstitial infiltrate in tissue samples—1309 cm^−1^ (CH_3_/CH_2_ twisting or bending mode of lipid & collagen), 1631 cm^−1^ (amide I), 1692 cm^−1^ (amide) (**D**) Wavenumber variables associated with interstitial fibrosis & tubular atrophy (IFTA) in paired urine samples—1247 cm^−1^ (amide III collagen assignment), 1175 cm^−1^ (cytosine, guanine), 932 cm^−1^ (proline, hydroxyproline), 1607 cm^−1^ (C=C phenylalanine, tyrosine) (**E**) Wavenumber variables associated with necrotising glomerular (GN) lesions in paired urine samples—1716 cm^−1^ (C=O of cortisone), 1316 cm^−1^ (guanine), 800 cm^−1^ (phosphate ion interactions) (**F**) Wavenumber variables associated with interstitial infiltrate in paired urine samples—1458 cm^−1^ (nucleic acid), 911 cm^−1^ (glucose).

**Figure 5:**
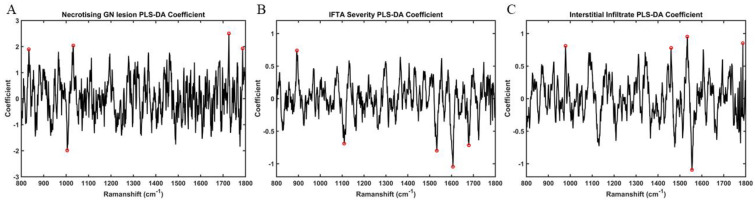
**PLS-DA coefficients for identification of spectral biomarkers from tissue samples (n = 27)**—(**A**) Wavenumber variables associated with necrotising glomerular (GN) lesions: 1726 cm^−1^ (C=O stretching vibrations of cortisone), 1031 cm^−1^ (C-H in-plane bending mode of phenylalanine), 833 cm^−1^ (asymmetric O-P-O stretching of tyrosine), 1787 cm^−1^ (C=O stretching vibrations of cortisone) (**B**) Wavenumber variables associated with interstitial fibrosis & tubular atrophy (IFTA): 893 cm^−1^ (phosphodiester deoxyribose) (**C**) Wavenumber variables associated with interstitial infiltrate: 1533 cm^−1^ (amide carbonyl group vibrations and aromatic hydrogens), 1787 cm^−1^ (C=O stretching vibrations of cortisone), 978 cm^−1^ (C-C stretching in β-sheet proteins), 1459 cm^−1^ (deoxyribose).

Based on PLS-DA coefficients, the key distinguishing spectral peaks and wavenumber assignments identified for necrotising glomerular lesions, interstitial infiltrate, and IFTA in the subgroup of paired tissue and urine samples are shown in Figure 4. Peaks associated with necrotising glomerular lesions in tissue were 1680 cm^−1^ (C=O, stretching vibrations of cortisone), 1443 cm^−1^ (CH2 bending mode of proteins & lipids CH2 deformation), 1539 cm^−1^ (amide carbonyl group vibrations & aromatic hydrogens) [18]. The only corresponding peak seen in urine was reflective of cortisone (1716 cm^−1^, C=O of cortisone), which is not specific to this type of lesion [18]. Although peaks representative of increased collagen deposition were seen in urine for IFTA, which would be anticipated (1247 cm^−1^, amide III collagen assignment), this was not observed in tissue [18]. Similarly, parallel biochemical activity for interstitial infiltrate was not seen between the two biosamples. Figure 5 demonstrates the key wavenumber variables for these same experimental classes amongst the overall cohort of 27 tissue samples.

## 4. Discussion

In this exploratory work, we demonstrate for the first time that biospectroscopy offers a potential novel method of machine learning with automated computational detection of AAGN disease activity in renal biopsy specimens. This was demonstrated with the ability of spectral data to distinguish the presence of histological lesions indicative of chronic damage and active disease with a high degree of accuracy, inclusive of IFTA, interstitial infiltrate, and necrotising glomerulonephritis.

Histological evaluation of renal biopsy samples remains the optimum method for diagnosing disease, but certain challenges remain. Key histological findings such as IFTA and interstitial infiltrate are potentially subject to inter-observer variability, with important prognostic implications of the former. As such, there remains scope for adjuvant techniques to complement and aid current tissue analysis. One evolving area of interest is machine learning. In a recent study, based on tissue staining with Masson trichrome and periodic acid-Schiff, Ginley et al. applied machine learning algorithms to digital images in order to reliably identify IFTA and glomerulosclerosis in cases of diabetic nephropathy and renal transplant specimens [19].

Biospectroscopy offers a means of extracting biochemical information that would not otherwise be accessible with current standard methods. By exploiting the interaction of light with the constituent molecules present within any given biosample, biospectroscopy has the capacity to generate a unique spectral fingerprint that is representative of the chemical bonds present. In doing so, the cellular activity unique to any given pathological state can be characterised. Two key analytical techniques are available, infrared spectroscopy and Raman spectroscopy. Both benefit from being low cost and label-free with minimal sample preparation required. Additionally, technological improvements and advancements in chemometric analysis over the past decade have enabled a high throughput of large datasets with increasing investigation of its potential application in renal medicine. In recent years, infrared spectroscopy has been successfully used to detect early biochemical variations that may precede histological changes seen in diabetic nephropathy amongst both native and transplant renal biopsy samples [20,21,22]. The same modality has also been applied in a large study by Vuiblet et al., to correctly quantify interstitial fibrosis and inflammation in renal transplant biopsies with >90% accuracy and good correlation with clinical outcomes [23]. Whereas the complementary method of Raman microspectroscopy has been investigated and validated as a viable technique to distinguish malignant renal tissue from both healthy parenchyma and benign disease [24,25], as well as successful tumour staging using spectra from surface-enhanced Raman scattering [26].

We applied Raman spectroscopy to unstained renal tissue samples from patients with histopathology reports consistent with AAGN. The resulting spectral data was able to correctly identify the presence of necrotising glomerular lesions, interstitial infiltrate and IFTA with a high degree of diagnostic accuracy on blind predictive modelling. The wavenumber-variables responsible for largest between group differences for the former two were associated with increased amino acid and cortisone activity, whereas IFTA tended to be associate with increased nucleic acid expression. The poor performance of the classification models according to the Berden classification system, presence of vessel wall necrosis. and the proportion of normal glomeruli likely reflects the limited sample distribution amongst these groups.

The aim of the subgroup analysis was to determine if the spectral data from urine could potentially be used as a surrogate for renal biopsy, the premise being that the biomolecular signature obtained from urine could characterise and reflect histological findings at a given time point. To address this question, the histological categories associated with good discriminatory function in the initial spectral analysis of the entire study cohort were evaluated in those tissue samples with a corresponding paired urine sample at the time of biopsy. These categories included necrotising GN lesions, interstitial infiltrate, and IFTA. Using the same chemometric methodology, there was limited performance in the model’s ability to reliably discriminate the presence of these categories in urine with a poor sensitivity in each group. This may have resulted from the limited sample size in each category amongst the subgroup and the possibility of insufficient training data. Taking this into account, it should not dissuade further research in this area. Excellent results have previously been obtained from the spectrochemical interrogation of other biofluids including plasma and serum, demonstrating both infrared and Raman spectroscopy as viable non-invasive candidate biomarker tools of disease activity in AAV [27,28]. Additionally, in the present study, it is worth noting that there was some similarity in the key distinguishing spectral peaks between the two biological samples, with increased protein and cortisone expression observed in both tissue and urine for necrotising glomerular lesions. As would be expected, notable biomolecular changes in urine for IFTA were representative of increased collagen synthesis, although this was not observed in tissue samples. One consideration is that the difference in the spectral acquisition method used for tissue and urine samples may account for the lack of consistency between spectral profiles obtained and the metabolic activities they represent. However, this would require a larger-sized and longitudinal temporal study to elucidate. It is also very possible that different profiles of spectral biomarkers present themselves depending on sample type.

Aside from sample size, one potential limiting factor is the absence of control groups. However, this is not essential herein with a factorial-based design determining the presence or absence of a feature in a cohort displaying a range of histological variation that is common amongst patients with AAGN. A further limitation is the lack of assigned variables to novel biomolecules reported in the literature. In our study, all experimental categories from tissue samples were based on known key histological variables. Promising non-invasive biomarkers of disease activity in AAGN include urinary monocyte chemoattractant protein-1 (uMCP-1), urinary soluble CD163 (sCD163), and degradation products of the complement cascade [29,30,31,32,33,34,35,36]. Each has been shown to correlate well with disease activity, in addition to an associated upregulation of macrophage infiltration in inflamed glomeruli with higher levels of uMCP-1 and the presence of fibrinoid necrosis and cellular crescents with sCD163 [29,37]. Tissue depositions of alternative pathway cleavage products including C3d, C3c, and Bb have been associated with a higher degree of cellular crescents and IFTA. This was mirrored in urine with higher levels of C3a, C5a, and soluble C5b-9 present in active disease, as well as higher urinary levels of Bb correlating with a lower proportion of normal glomeruli [36]. Any future study evaluating the role of biospectroscopy in AAGN would benefit from assay and analysis of these potential biomarkers with spectral data. In addition to offering a potentially cheaper and faster surrogate technique for their detection, their analysis may also help resolve the current lack of concordance in the spectral profiles between the two biosamples. Other potential areas of research include the application of forward feature extraction algorithms to construct prediction outcome models based extracted spectral features, as well as correlation of spectral data with imaging mass spectrometry to aid in the delineation of any potential biomarkers.

Vibrational spectroscopy has the potential to offer a robust tool for machine learning and standardised automated detection of disease activity in AAGN. Its application enables the simultaneous analysis of a broad spectrum of biomolecules, providing an adjuvant technique for biomarker extraction and additional potential insight into the molecular mechanism of disease. This study highlights the potential for spectral profiles to be used as a non-invasive surrogate marker of histological changes in order to aid disease monitoring and guide patient care. The role of biospectroscopy in tissue warrants further research in a larger study of varied renal pathology, with comparison alongside biofluids to determine its plausible use as a liquid biopsy.

## Figures and Tables

**Table 1:** Characteristics of study population.

	AAGN (n = 27)
**Mean Age (SD)**	63 ± 10
**Sex**	
Male Female	15/27 (55.6%) 12/27 (44.4%)
**Median serum creatinine at biopsy (** **μmol/L)**	215 (338–164)
**Median eGFR at biopsy (mls/min/1.73 m^2^)**	22 (33–12)
**ANCA serotype at biopsy**	
MPO	12/27 (44.4%)
PR3	12/27 (44.4%)
Negative	3/27 (11.1%)
**Mean number of glomeruli per biopsy sample**	
**Berden classification**	20 ± 9
Focal	15/27 (55.6%)
Crescentic	3/27 (11.1%)
Sclerosed	0
Mixed	9/27 (33.3%)
**Normal glomeruli**	
N0 (>25%)	21/27 (77.8%)
N1 (10–25%)	2/27 (7.4%)
N2 (<10%)	4/27 (14.8%)
**IFTA**	
T0 (≤25%)	20/27 (74.1%)
T1 (>25%)	7/27 (25.9%)
**Necrotising glomerular lesions**	16/27 (59.3%)
**Interstitial infiltrate**	10/27 (37%)
**Extra-glomerular arteritis**	5/27 (18.5%)
**Vessel wall necrosis**	4/27 (14.8%)

ANCA, anti-neutrophil cytoplasmic autoantibody; MPO, myeloperoxidase; PR3, proteinase-3; IFTA, interstitial fibrosis & tubular atrophy.

**Table 2:** Classification model performance according to histological data for renal biopsy samples (n = 27).

Presence of Histological Features as an Experimental Class	Best Discriminate Model	Spectral Data	Accuracy (%)	Sensitivity (%)	Specificity (%)	F-Score (%)	G-Score (%)
**Berden classification: Focal vs. Mixed**	PLS-DA (3 LVs)	Training: TPS	81	82	78	80	80
		CV: TPS	75	80	67	73	73
		Test: MPS	69	69	70	69	69
**Normal Glomeruli:** **N0 vs. N1&N2**	PLS-DA (3 LVs)	Training: TPS	90	89	90	89	89
		CV: TPS	77	56	83	67	68
		Test: MPS	93	83	95	89	89
**IFTA: T0 vs. T1**	PLS-DA (3 LVs)	Training: TPS	91	86	93	89	89
		CV: TPS	78	67	82	74	74
		Test: MPS	93	100	90	95	95
**Necrotising glomerular lesions**	PLS-DA (8 LVs)	Training: TPS	100	100	100	100	100
		CV: TPS	87	88	85	86	86
		Test: MPS	100	100	100	100	100
**Interstitial Infiltrate**	PLS-DA (3 LVs)	Training: TPS	88	87	88	87	87
		CV: TPS	80	73	84	78	78
		Test: MPS	100	100	100	100	100
**Extra-glomerular arteritis**	PLS-DA (2 LVs)	Training: TPS	74	80	73	76	76
		CV: TPS	72	67	73	70	70
		Test: MPS	78	100	73	84	85
**Vessel Wall Necrosis**	PLS-DA (2 LVs)	Training: TPS	74	92	71	80	81
		CV: TPS	69	58	71	64	64
		Test: MPS	81	100	78	88	88

Berden classification, Focal: ≥50% normal glomeruli, Mixed: no predominant lesion; Normal glomeruli, N0 > 25%, N1 10–25%, N2 < 10%; IFTA, interstitial fibrosis & tubular atrophy, T1 > 25%, T0 ≤ 25%; PLS-DA, partial least squares discriminant analysis; LVs, latent variables; TPS, total processed spectra; MPS, mean processed spectra, CV; cross-validation.

**Table 3:** Classification model performance according to histological data: comparative results for paired tissue & urine samples (n = 10).

Presence of Histological Features as an Experimental Class	Sample	Best Discriminate Model	Spectral Data	Accuracy (%)	Sensitivity (%)	Specificity (%)	F-Score (%)	G-Score (%)
**Presence of IFTA: T0 vs. T1**	Tissue	PLS-DA (6 LVs)	Training: TPS	100	100	100	100	100
			CV: TPS	83	67	90	77	78
			Test: MPS	100	100	100	100	100
	Urine	PLS-DA (10 LVs)	Training: TPS	100	100	100	100	100
			CV: TPS	66	57	70	63	63
			Test: MPS	100	100	100	100	100
**Presence of Necrotising glomerular lesions**	Tissue	PLS-DA (3 LVs)	Training: TPS	100	100	100	100	100
			CV: TPS	87	78	90	84	84
			Test: MPS	100	100	100	100	100
	Urine	PLS-DA (4 LVs)	Training: TPS	93	93	93	93	93
			CV: TPS	67	53	73	61	62
			Test: MPS	100	100	100	100	100
**Presence of Interstitial Infiltrate**	Tissue	PLS-DA (6 LVs)	Training: TPS	100	100	100	100	100
			CV: TPS	90	89	90	89	89
			Test: MPS	100	100	100	100	100
	Urine	PLS-DA (10 LVs)	Training: TPS	99	97	100	98	98
			CV: TPS	72	53	80	64	65
			Test: MPS	100	100	100	100	100

IFTA, interstitial fibrosis & tubular atrophy, T1 > 25%, T0 ≤ 25%; PLS-DA, partial least squares discriminant analysis; LVs, latent variables; TPS, total processed spectra; MPS, mean processed spectra, CV; cross-validation.

## Data Availability

The authors declare that the data supporting the findings of this study are available within the paper and its supplementary information. The MATLAB code and instructions on how to process the data are presented in previous publications (13, 14, 17).
